# Prevalence of Virulence/Stress Genes in *Campylobacter jejuni* from Chicken Meat Sold in Qatari Retail Outlets

**DOI:** 10.1371/journal.pone.0156938

**Published:** 2016-06-03

**Authors:** Marawan Abu-Madi, Jerzy M. Behnke, Aarti Sharma, Rebecca Bearden, Nadia Al-Banna

**Affiliations:** 1 Department of Biomedical Science, Biomedical Research Center, College of Health Sciences, Qatar University, P.O. Box 2713, Doha, Qatar; 2 School of Life Sciences, University of Nottingham, University Park, Nottingham, United Kingdom NG7 2RD; 3 College of Medicine, Qatar University, P.O. Box 2713, Doha, Qatar; Massey University, NEW ZEALAND

## Abstract

Chicken meat from the shelves of supermarkets in Qatar was tested for the presence of *Campylobacter* spp. and the presence of five virulence genes (*htrB*, *cdtB*, *clpP*, *cadF* and *ciaB*) was assessed in isolates. Forty eight percent of the chickens provided for supermarkets by Saudi (53%) and Qatari (45.9%) producers were found to be contaminated and the most important factor affecting the overall prevalence of contaminated chickens was the store from which chicken samples originated. Variation in prevalence of *Campylobacter* in chicken meat from different stores was evident even when the same producer supplied the three stores in our survey. Differences in the prevalence and in the combinations of virulence genes in isolates that can and cannot grow in a classic maintenance medium (Karmali) were identified, providing a starting point for linking presence/absence of particular virulence genes with actual *in vivo* virulence and pathogenicity. Because of the relatively low infective doses of *Campylobacter* that are required to initiate infection in humans, it will be important to explore further the relationships we identified between certain *Campylobacter* virulence genes and their capacity for survival in poultry meat, and hence their contribution to the incidence of campylobacteriosis.

## Introduction

*Campylobacter* is the leading cause of bacterial gastroenteritis in the world [1]. Infection is generally manifested as self-limiting diarrhoea that lasts 3–5 days, but in some cases it may progress to bloody diarrhoea and have life-threatening consequences. *Campylobacter* is suspected of causing disease via cytotoxin production [2], intestinal cell invasion [3] and extra-intestinal adherence and translocation [4]. Several genes have been linked to *Campylobacter* virulence but the most important are *Campylobacter* invasion protein B (*ciaB*), which is vital for invasion, cytolethal distending toxin B (*cdtB*), which disrupts mucosal barriers by causing host cell death, *Campylobacter* adhesin to fibronectin F (*cadF)*, and the heat survival and stress response proteins *htrB* and *clpP*, which are important for survival [5,6].

Most human campylobacteriosis cases are caused by a single species, *Campylobacter jejuni*, which makes it clinically and microbiologically the most relevant species. *Campylobacter* is ubiquitous and has been isolated from water, milk, and beef. The main sources of contamination are via the handling and consumption of fresh and undercooked poultry products, in domestic settings, restaurants and in commercial facilities [7]. An Iranian study found that *Campylobacter* contamination was more commonly found among raw chicken meat than beef collected at retail stores in Tehran [8]. The percentage of contaminated poultry carcasses varies from country to country, it can be as low as 15%, as in Iceland [9], and as high as 90%, as in the USA [10].

Several studies have emphasized the direct role that poultry consumption plays in the spread of *Campylobacter*. During the early summer of 1999, the dioxin crisis started in Europe when the carcinogenic substance was found in the feed of poultry. A ban was issued and all poultry products were withdrawn and banned from the market. During this period there was a 40% reduction in the number of reported *C*. *jejuni* infections [11]. Another line of evidence comes from Iceland which, prior to 1996, had a low incidence of *C*. *jejuni* infections [9]. However, after 1996, fresh poultry products were introduced into the markets, and the incidence of *C*. *jejuni* confirmed cases increased from only 38 cases per 100,000 to reach a peak of 116 cases per 100,000 in 1999. Upon investigation, it was found that 62% of broiler carcasses were contaminated with *C*. *jejuni* [9]. As a preventive measure, birds from poultry flocks testing positive were frozen and only birds from *C*. *jejuni*-free flocks were sold fresh, and subsequently, there was a drop of 16% in the availability of fresh chicken carcasses contaminated with *C*. *jejuni*. The corresponding drop in human cases was significant, with only 33 cases per 100,000 in 2000 [9]. These two examples indicate that poultry products are responsible for a significant proportion of human infections. The data collected through a *Campylobacter* screening study in The Netherlands further supports the hypothesis that poultry is a critical risk factor for *Campylobacter* infection. This study estimated that 20–40% of laboratory-confirmed *C*. *jejuni* cases were linked to poultry consumption or handling [12].

Although campylobacteriosis is a well-recognized problem in Qatar [13] and other countries of the Gulf Cooperation Council, there are no surveillance programs for *Campylobacter* in the region [14]. While many developed countries have routine procedures for the inspection of poultry products for the presence of *Campylobacter* no such procedures have been implemented in Qatar. Therefore, in this study, we collected fresh whole chickens of variable weight and from a range of producers and retailers in Qatar. We then assessed the degree to which these three factors (producer, retailer and weight of carcass) affect the prevalence of the bacterium on chicken carcasses. We also determined the percentage of *C*. *jejuni* isolates having virulence factors that might contribute to human infection and colonization of chickens. The availability of such information should allow for better local policy drafting by food safety and public health officials and will increase awareness of this medically and economically important bacterium in Qatar and more widely in the Middle East.

## Materials and Methods

### Isolation and identification of *Campylobacter spp*.

Ethical approval was obtained from the Institutional Animal Care and Use Committee of Qatar University with number QU-IACUC 002/2011, and this required anonymity being maintained with respect to the identity of the sources of chickens for the study. For this reason, in the analyses that follow we used numbers to distinguish between companies and stores. The sampled chickens were raised by eight different producers, numbered 1–8, and were purchased from three stores in Qatar. One of the producers was based in Qatar (company no. 1). Five of the other companies were identified as being based in Saudi Arabia but for two (no. 7 and 8) we were not able to trace where their chickens were raised.

A total of 400 packages were purchased over a period of one year. Purchased samples were transported in an icebox to maintain the temperature at +4°C. Samples were processed within 1 hour of purchase to ensure maximum recovery of *Campylobacter*. Upon arrival in the laboratory, samples were opened aseptically and 25 skin samples were collected from the neck, breast and back areas, avoiding the fat. The skin was rinsed with 100ml of ice-cold buffered peptone water (BPW) (Difco, Sparks, MD, USA) and placed along with the BPW, for an enrichment step, in a sterile filter Stomacher plastic bag. The bag was vigorously pounded on its outer surfaces by paddles at 250rpm for 90s before discarding the filter along with the skin. A gas mixture (85% N_2_, 10% CO_2_, 5% O_2_) was infused to create microaerophilic conditions inside the bag, which was finally sealed with a thermo-sealer and incubated for 48 hrs at 42°C. The rinse was centrifuged, re-suspended, and a sample of 25μl was plated on Karmali *Campylobacter* agar (Oxoid Cat# CM0935) at 42°C under microaerophilic conditions for 48 hrs. Growing colonies were subsequently confirmed to be *Campylobacter* by Real Time (RT)-PCR (below). For each RT-PCR batch, a confirmed *C*. *jejuni* strain (ATCC 33560) was used as a positive control. A subculture from each positive sample was grown to provide material for the identification of virulence genes and this was stored at -70°C until needed.

### DNA extraction

DNA was extracted from freshly grown colonies on Karmali plates using Mericon DNA Bacteria Kits (Qiagen, D-40724 Hilden, Germany). In brief, colonies from Karmali agar plates were suspended in 1000μl of water and centrifuged at 13000 × g for 5min. The pellet was suspended in 400μl of lysis buffer (provided in the kit), vortexed and incubated at 95°C for 10 min. Samples were allowed to cool, centrifuged at 13000 × g for 5min and then 100μl of each sample was transferred to 1.5ml microcentrifuge tubes and stored for future use at -20°C. The quality and quantity of the DNA was determined using Nanodrop (Qiagen, Germany). The same procedure was also used to extract DNA from BPW enrichment media.

### Real Time (RT)-PCR detection

RT-PCR was performed using the following primers Forward 5’ CTATAACAACTGCACCTACTAT 3’, Reverse 5’ATGAAATTTTTGCCAGTGGTG 3’ and Probe 5’ FAM/CTTAATAGCCGTCACCCCAC 3 for *C*. *jejuni* detection and Fwd 5’ GGTATGAAAACTACAAAGCGAG3’, Rvs 5’ ATATTTAGACTATCGTCGCGT3’ for *C*. *Coli* identification. The primers were tested on *C*. *jejuni* (ATCC 33560) and *C*. *coli* (ATCC 33559) to confirm their efficacy. A total of 7μl of Syberselect (Life Technologies) master mixture was used in each reaction along with 0.3μl primer mixture, 2.5μl sample DNA and 10.2μl of molecular grade water. RT-PCR consisted of incubating for 2 min at 95°C followed by 40 cycles of 95°C for 15s, 55.5°C for 35s and 72°C for 1min. Samples were confirmed as positive by comparing to the standard curve of the positive control.

### Potential virulence factors genes

RT-PCR was used to detect the presence of five genes (*ciaB*, *cadF*, *cdtB*, *htrB* and *clpP*), which are involved mainly in adhesion and invasion [15]. These five genes will be referred to as virulence factors from this point onwards. The primers used and the RT-PCR protocol, were as described in our previous work with *Campylobacter* [13]. Samples having positive Ct values were confirmed initially by agarose gel electrophoresis for each of the genes targeted in this study. Specimens were run in duplicates.

### Statistical analysis

Altogether 400 chicken carcasses obtained from supermarket stores in Qatar were sampled, and the overall prevalence is based on this number. However, statistical analyses of *C*. *jejuni* were based on *n* = 382. Some companies were eliminated from the analysis if the sample sizes for the suppliers were small (Company no. 5, *n* = 3 and Company no. 8, *n* = 5) or if information regarding the country in which the chickens were produced proved to be unreliable (Company no. 7, *n* = 10 and Company no. 8). The sampled chickens weighed between 500 and 1300 g. For convenience they were allocated to three weight classes as follows (weight class 1 = 500 to 800g; weight class 2 = >800 to 1000g; weight class 3 = >1000 to 1300g).

The results are presented as percentages with 95% confidence limits (CL_95_), calculated with bespoke software based on the tables of Rohlf and Sokal [16]. For analysis we used maximum likelihood techniques based on log linear analysis of contingency tables in the software package IBM SPSS Statistics Version 21 (IBM Corporation). Initially, full factorial models were fitted, incorporating as factors COMPANY (5 levels), STORE (3 levels), and WEIGHT CLASS (3 levels). The presence / absence of *Campylobacter* (INFECTION) was fitted as a binary factor. These explanatory factors were fitted initially to all models that were evaluated. For each level of analysis in turn, beginning with the most complex model, involving all possible main effects and interactions, those combinations that did not contribute significantly to explaining variation in the data were eliminated in a stepwise fashion beginning with the highest level interaction (backward selection procedure). A minimum sufficient model was then obtained, for which the likelihood ratio of χ^2^ was not significant, indicating that the model was sufficient in explaining the data. The importance of each term (i.e. interactions involving INFECTION) in the final model was assessed by the probability that its exclusion would alter the model significantly and these values relating to interactions that included INFECTION are given in the text. Where complex three way interactions were retained as significant in the final model, we explored these in figures, and also assessed the importance of each of the main effects in turn, fitting simpler models based only on INFECTION and the factor in question (referred to as single factor model). For analysis of numeric scale data, we used the Mann-Whitney U test.

## Results

### Prevalence of *C*. *jejuni* among chicken skin sampled from Qatari Markets

We investigated the prevalence of the bacterium on 400 chicken carcasses. Skin samples of 400 chickens were subjected to the BPW enrichment step then extracted and cultured on Karmali plates. Of the 400 chickens tested, samples from 36.5% (CL_95_ = 30.76–42.61, *n* = 146) generated colonies in Karmali agar.

DNA was extracted from grown colonies (i.e. “Karmali-agar positive samples”). Identification of *C*. *jejuni* was confirmed by RT-PCR. Of the chickens tested, 80.1% (CL_95_ = 72.68–86.11, *n* = 117) of the Karmali-agar positive samples were confirmed as *C*. *jejuni* by specific RT-PCR. Only one of the chickens was found to be contaminated with *C*. *coli* and this was a mixed contamination since *C*. *jejuni* was also found on this chicken. DNA extracted from the BPW enrichment media of 24 of the 29 *C*. *jejuni* negative samples was also tested for *C*. *jejuni* with the specific PCR probe and all proved negative.

In addition, 254 chicken skin extracts that did not produce colonies in Karmali agar were then tested for evidence of *C*. *jejuni* in the DNA extracted from the BPW enrichment media. Seventy-five samples were positive for *C*. *jejuni* DNA (29.5%, CL_95_ = 25.25–34.17).

**Fig 1:**
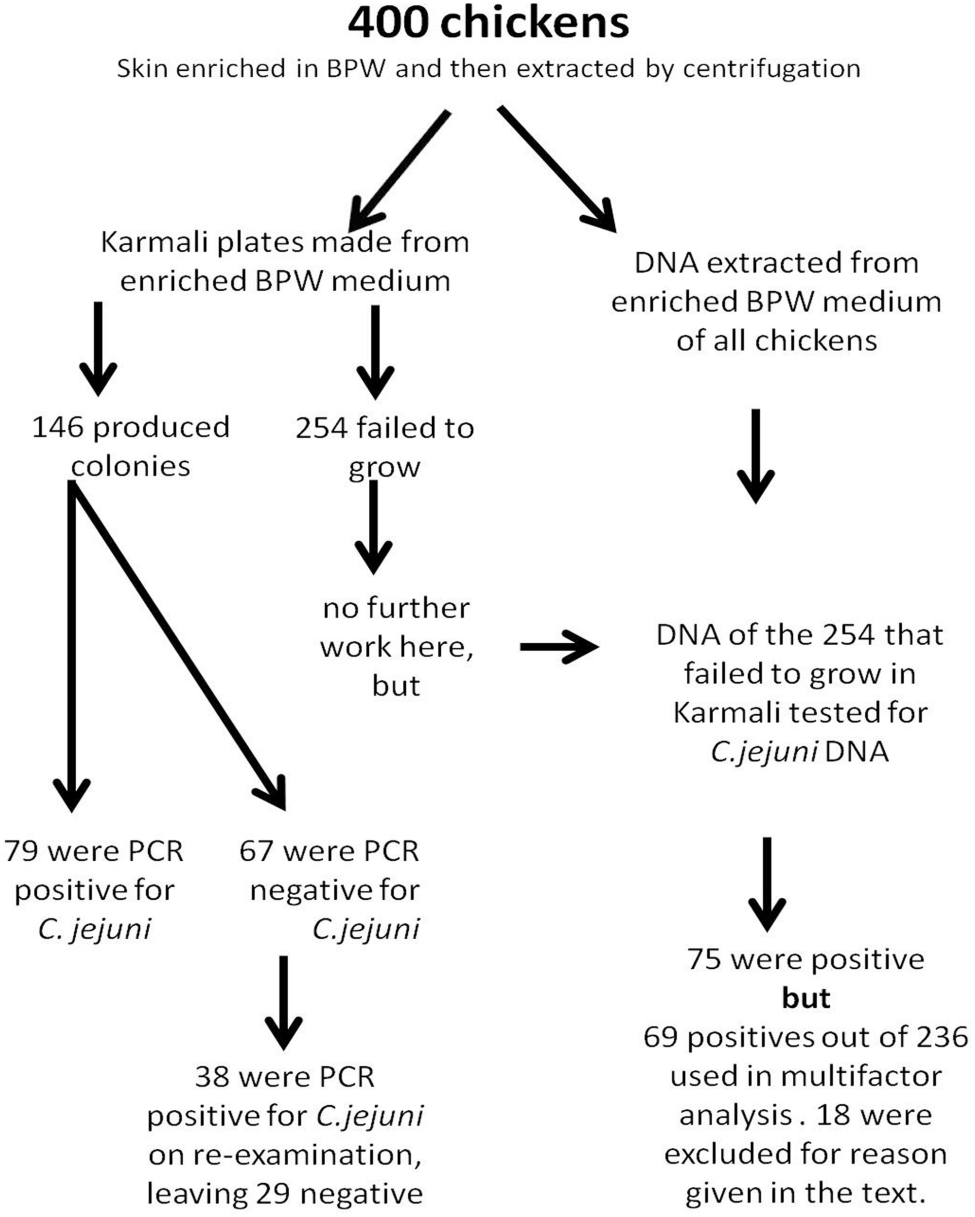
Schematic diagram of the stages in the analysis of samples. The number of samples at each stage are shown.

The samples that were positive for *C*. *jejuni* from the Karmali agar plates were combined with the additional 75 that proved positive by DNA extracted directly from the BPW enrichment media to give a total of 192 chickens positive for *C*. *jejuni* (48.0%, CL_95_ = 41.86–54.13). The results from the analysis of the 400 chicken samples are summarized in Fig 1.

### Factors affecting the prevalence of *Campylobacter* spp.

**Fig 2:**
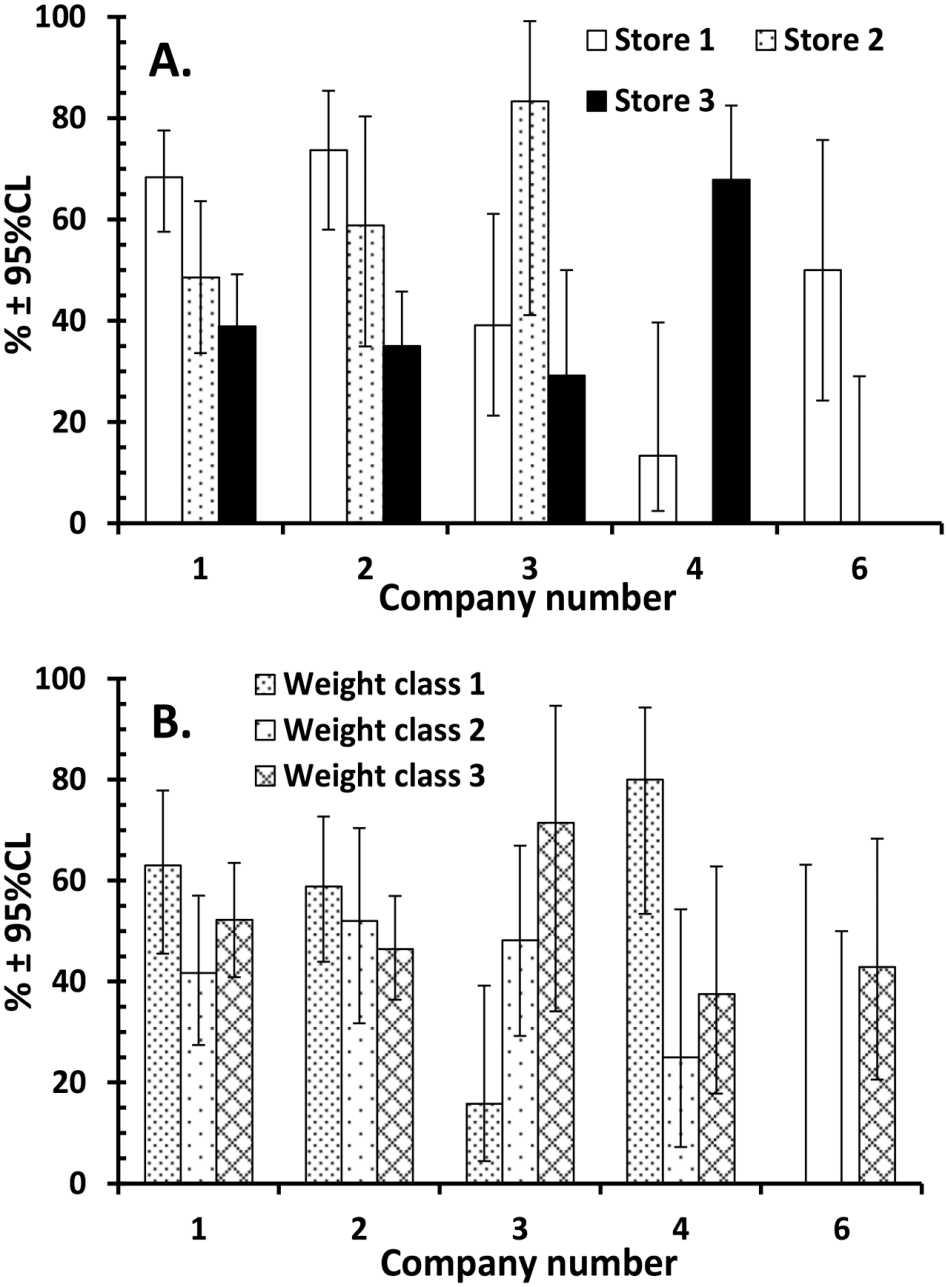
Factors affecting the percentage of contaminated chickens. (A) Variation in the percentage of contaminated chickens from producer companies detected at three stores; (B) Variation in the percentage of contaminated chickens among the three weight classes from the various producer companies.

Further statistical analysis was confined to 382 samples (from companies 1,2,3,4 and 6) since, as explained in the Materials and Methods section, for three producer companies (ref. numbers 5,7 and 8) we had either small sample sizes or unknown locations of producers or both. Analysis by a full factorial model revealed two interesting interactions between factors and these are illustrated in Fig 2. There was significant variation in the percentage of contaminated chickens purchased between the three stores but derived from the same producing company (Fig 2A; producer company x store x INFECTION *χ*^2^_8_ = 31.4, *P* < 0.001; See for example store 3). There was also significant variation between the producers in the percentage of contaminated chickens among the three weight classes (Fig 2B; producer company x weight class x INFECTION *χ*^2^_8_ = 21.2, *P* = 0.007). The percentage of contaminated chickens in different weight classes from Producer Company 1 for example, was similar, whereas for Producer Company 3 the percentage of contaminated chickens increased significantly with weight, and Producer Company 4 supplied most contaminated chickens in the lightest weight class.

Table 1 shows the percentage of chicken carcasses contaminated with *C*. *jejuni* by country in which the poultry producer was based, company producing the chickens, store providing the chicken, and weight class of chicken. When tested in single factor models, only one of these main effects was associated with significant variation in the percentage of contaminated chickens (Table 1, stores differed significantly in the percentage of contaminated chickens they supplied).

**Table 1:** Prevalence (%) of *C*. *jejuni* by country in which the producer was based, the company providing chickens, the store selling the chickens and the weight class of samples chickens.

Factor	Level*	*N*	%	CL_95_	DoF	χ^2^	*P*
Country	Qatar	149	53.0	44.54–61.29			
	Saudi Arabia	233	45.9	41.24–50.61	1	1.83	0.176
Company	1	149	53.0	44.54–61.29			
	2	115	51.3	43.85–58.76			
	3	53	39.6	30.10–49.86			
	4	43	48.8	32.63–65.42			
	6	22	27.3	12.61–50.00	4	7.40	0.116
Store	1	148	58.1	49.67–66.22			
	2	68	47.1	35.96–58.54			
	3	166	32.35	32.35–49.94	2	9.33	**0.009**
Weight class	1	117	54.7	47.19–62.00			
	2	105	41.9	35.00–49.05			
	3	160	48.8	39.95–57.57	2	3.64	0.162

The sample size was *n* = 382

* See Materials and methods for further information about each of the factor levels.

### Virulence factors

The percentage of isolates that grew on Karmali agar plates possessing the five virulence factors is shown in Table 2. Data are included for both the *C*. *jejuni* PCR-positive and -negative samples. As can be seen, all five virulence factors were present in both *C*. *jejuni*-positive and negative isolates. In positive cultures a prevalence of more than 94% was recorded in each case, whereas in the *C*. *jejuni*-negative cultures prevalence was lower. Testing by 2 x 2 Chi Squared indicated that in four cases prevalence was significantly higher in the positive cultures, however, the gene *ciaB* was universally detected in all isolates. These data were fitted into a multifactorial model and only two of the virulence factors retained significance (*htrB χ*^2^_1_ = 3.86, *P* = 0.049 and *clpP χ*^2^_1_ = 7.43, *P* = 0.006).

**Table 2:** Presence of 5 virulence factors in colonies grown in Karmali medium that were either positive or negative for *C*. *jejuni*, and combined.

Virulence factor	Percentage (CL_95_)		
	*C*. *jejuni* +ve (*n* = 117)	*C*. *jejuni* -ve (*n* = 29)	Combined (*n* = 146)
*htrB*	94.9 (90.50–97.34)	75.9 (57.00–88.46)*	91.1 (85.06–94.89)
*cdtB*	94.9 (90.50–97.34)	72.4 (53.50–86.39)**	90.4 (84.35–94.38)
*clpP*	99.2 (96.33–99.86)	82.8 (64.05–92.95)***	95.9 (91.17–98.27)
*cadF*	96.6 (92.71–98.53)	75.9 (57.00–88.46)****	92.5 (86.73–95.89)
*ciaB*	100 (97.81–100)	100 (88.47–100)	100 (97.27–100)

* *χ*^2^_1_ = 8.74, *P* = 0.0031

** *χ*^2^_1_ = 11.73, *P* = 0.0006

*** *χ*^2^_1_ = 12.11, *P* = 0.0005

**** *χ*^2^_1_ = 12.23, *P* = 0.0005

However, the model revealed interesting and significant interactions between some of the virulence factors, and these are summarised in Table 3. Cultures that were positive for *cadF* were almost universally also positive for *clpP*, and those positive for *cdtB* showed a high prevalence also of *clpP* and *htrB*.

**Table 3:** Significant interactions among virulence factors in isolates that grew in Karmali medium (*n* = 146).

	%	CL_95_	χ^2^	*P*
*cadF* negative	*clpP* = 54.5	(26.46–80.04)		
*cadF* positive	*clpP* = 99.3	(96.21–99.90)	23.1	<0.001
*cdtB* negative	*clpP* = 64.3	(37.11–84.72)		
*cdtB* positive	*clpP* = 99.2	(96.23–99.89)	15.6	<0.001
*cdtB* negative	*htrB* = 42.9	(20.61–68.28)		
*cdtB* positive	*htrB* = 96.2	(91.91–98.40)	21.6	<0.001

Almost all the *C*. *jejuni*-positive colonies possessed all five virulence factors (104/117 = 88.9%) and there was just one colony that had a single virulence factor (*ciaB*). In contrast, only 17/29 *C*. *jejuni*-negative colonies (58.6%) had all five virulent factors and 3 had just one (again *ciaB* in each case).

### Karmali-negative samples and analysis of BPW enrichment media isolates

Among the 75 samples that were positive for *C*. *jejuni* DNA (29.5%), each of the virulence factors was detected far less frequently than in the samples that grew successfully in Karmali medium (Table 4). Only 5/75 (6.7%) of DNA samples had all 5 virulence factors, while 43 (57.3%) had none and 17 (22.7%) just one of the five.

**Table 4:** Presence (%) of 5 virulence factors in DNA samples from BPW enrichment media of chickens that failed to produce colonies in Karmali medium.

Virulence factor	%	(CL_95_)
*htrB*	12.0	(6.12–21.86)
*cdtB*	6.7	(2.57–15.38)
*clpP*	30.7	(20.66–42.63)
*cadF*	26.7	(17.27–38.36)
*ciaB*	8.0	(3.27–17.07)

### Comparison of the number of virulence factors in samples that were positive and negative for *C*. *jejuni* in Karmali medium and positive for *C*. *jejuni* in the BPW enrichment medium

The mean number of virulence factors detected in samples grown in Karmali medium that were positive for *C*. *jejuni* was 4.86 ± 0.046 (*n* = 117), and those that were negative 4.07 ± 0.258 (*n* = 29; Combined = 4.7 ± 0.07, *n* = 146). Although the mean difference is small, nevertheless it was highly significant (Mann-Whitney *U* test, *z* = 4.1, *P* < 0.001).

These values compare with a mean of just 0.84 ± 0.155 (*n* = 75) among the *C*. *jejuni*-positive DNAs extracted from the BPW enrichment media of those chickens that failed to produce colonies in Karmali medium. The difference in the number of virulence factors detected in the DNA of *C*. *jejuni*-positive colonies in Karmali medium and the DNA from BPW enrichment media of those chickens that failed to provide colonies, but were positive for *C*. *jejuni* by PCR, was hugely significant (Mann-Whitney *U* test, *z* = 12.0, *P* < 0.001).

### Comparison of the number of virulence factors in chickens supplied by Qatari and Saudi Arabian producers

Table 5 shows the prevalence of the virulence factors in samples from chickens obtained from Qatari and Saudi Arabian producers. The data include typing for virulence factors on bacteria grown on Karmali medium that were positive for *C*. *jejuni* by PCR, and those that were not, and also on DNA obtained from BPW enrichment media. It can be seen that the prevalence of the virulence factors *cdtB* and *clpP* was lower in isolates obtained from Saudi Arabian producers, but there were no other significant differences between isolates derived from chickens from the two countries.

**Table 5:** Comparison of prevalence (%) of virulence factors on chickens supplied by Qatari and Saudi Arabian producers.

Factor	Source	PCR for Cj	Qatar % (CL_95_)	Saudi Arabia %(CL_95_)	*χ*^2^*	P*
*htrB*	Karmali	+ve	92.6 (85.24–96.57)	96.8 (90.39–99.19)	1.078	0.299
*htrB*	Karmali	-ve	80.0 (34.26–98.97)	75.0 (54.24–88.50)	0.058	0.809
*htrB*	Karmali	combined	91.5 (83.51–96.04)	90.8 (80.48–96.16)	0.023	0.880
*cdtB*	Karmali	+ve	98.1 (92.74–99.70)	92.1 (83.85–96.52)	2.443	0.118
*cdtB*	Karmali	-ve	80.0 (34.26–98.97)	70.8 (50.00–86.08)	0.184	0.668
*cdtB*	Karmali	combined	96.6 (90.37–99.05)	86.2 (74.66–93.01)	**4.984**	**0.026**
*clpP*	Karmali	+ve	100 (95.95–100)	98.4 (92.61–99.80)	1.245	0.264
*clpP*	Karmali	-ve	100 (50.00–100)	79.2 (58.49–91.41)	2.099	0.147
*clpP*	Karmali	combined	100 (95.58–100)	93.1 (83.38–97.55)	**6.386**	**0.012**
*cadF*	Karmali	+ve	96.3 (90.28–98.83)	96.8 (90.39–99.19)	0.025	0.875
*cadF*	Karmali	-ve	60.0 (18.93–92.35)	79.2 (58.49–91.41)	0.761	0.383
*cadF*	Karmali	combined	93.2 (85.75–97.01)	92.0 (82.04–96.93)	0.082	0.775
*htrB*	BPW	+ve	8.0 (1.45–25.59)	10.9 (3.55–26.31)	0.155	0.694
*cdtB*	BPW	+ve	4.0 (0.21–19.56)	6.5 (1.31–20.39)	0.204	0.651
*clpP*	BPW	+ve	36.0 (19.57–56.08)	26.1 (13.32–43.48)	0.753	0.386
*cadF*	BPW	+ve	20.0 (8.23–39.84)	28.3 (15.11–45.68)	0.599	0.439
*ciaB*	BPW	+ve	8.0 (1.45–25.59)	6.5 (1.31–20.39)	0.053	0.818

For Karmali isolates for +ve, -ve and combined isolates from Qatar the sample sizes were 54, 5 and 59, and for Saudi Arabia 63, 24 and 87, respectively.

For DNA isolated from BPW enrichment media, all positive for *C*. *jejuni*, the sample sizes were 25 from Qatar and 46 from Saudi Arabia

* Significant outcomes are emphasized in bold. All tests have one degree of freedom.

## Discussion

*Campylobacter* has been established as the leading cause of bacterial gastroenteritis worldwide, and with an estimated 2–28% of diarrhoeal cases attributed to these bacteria in the Arabian Gulf countries [17, 18, 19, 20]. The most common route of transmission is via consumption of contaminated poultry products and our data show clearly that a significant percentage (48%) of the chickens available on the shelves of supermarkets in Qatar carry these bacteria. This is consistent with other studies on the contamination of chicken carcasses with *C*. *jejuni* in the region: for example 52.25% of chickens in Saudi Arabia [21], 57% from a study in Bahrain [22], and between 36.5%-76% from various studies in different parts of Iran [8, 23, 24, 25, 26]. Worldwide *C*. *jejuni* is responsible for 85% of cases of human campylobacteriosis and is the most frequently isolated *Campylobacter* species recovered from poultry [26, 27, 28], while the remaining cases are primarily attributed to *C*. *coli* [29]. This is despite over 30 species being known to exist, most of the other species being parasites of other warm-blooded animals [30, 31]. In our study, only one sample proved to be *C*. *coli* based on specific primers that we used, and this is consistent with other studies [32].

In the present study, the chickens came from both Saudi and Qatari producers but the prevalence of *C*. *jejuni* was similar, 53% and 45.9%, respectively. While relatively consistent across Middle-Eastern countries, the prevalence of *Campylobacter* spp. in chickens in the region is still quite different to values reported from the USA where prevalences exceeding 90% have been reported on chicken carcasses [10].

In the current work, 36.5% of the 400 samples that were tested showed good colony growth on selective Karmali agar, indicating that these could be *Campylobacter* spp., and 80.1% of these were confirmed as *C*. *jejuni* by PCR. However, some colonies grown on Karmali plates were not amplified using our *C*. *jejuni* or *C*. *coli*-specific PCR probes and therefore could not be confirmed as either of these two species. This may be due in part to the fact that Karmali agar, although developed for the isolation of *Campylobacter* spp., can give a positive red colouring for other non-*Campylobacter* strains [33, 34]. It is also possible that the negative PCR results can be attributable to genetic variations in these isolates, such as point mutations in the regions complementary to our primer sequence, thereby altering binding by our primers and preventing amplification. This is an important limitation of the molecular methods employed here, which rely entirely on the amplification of the targeted region.

In order to increase the accuracy of our approach, our strategy also comprised detection of *Campylobacter* in the BPW enriched media for the 254 samples that did not produce colonies on Karmali agar, as BPW has been shown to yield higher detection rates for *Campylobacter* in PCR [35]. Among these samples, we found evidence for the presence of *C*. *jejuni* in a further 29.5% of chickens. The fact that we detected *C*. *jejuni* DNA in such samples may reflect the capacity of *Campylobacter* to survive in cold environments for long periods of time (e.g. +4°C on the skin of chickens), resulting in the final poultry products that are sold to the consumer being contaminated with the bacteria, despite refrigeration. When subjected to unfavourable conditions, such as cold exposure, *Campylobacter* spp. have been shown to enter a viable but non-culturable (VBNC) state, in which the bacteria are not able to grow in classic media that normally support their growth [36]. However, they remain viable and may revert to virulence when introduced into the gastrointestinal tract of hosts [37, 38]. RT-PCR methods, using propidium monoazide, have been described for the quantification of viable *Campylobacter*, including those in the VBNC state [39], and thus DNA can be detected in colony-negative samples.

Variation in the prevalence of *Campylobacter* spp. was analysed statistically to identify any interactions between the producer and retailer of the samples under investigations (Table 1) and the size of the chicken carcasses. The most important factor affecting the overall prevalence was the store from which chicken samples originated, and variation in prevalence between stores was evident even when the same producer supplied the three stores in our survey. This suggests that local conditions at the supermarkets are an important contributory factor, either enhancing or inhibiting contamination with *Campylobacter* during preparation, storage, or display of chicken carcasses in the sale halls of supermarkets. Temperature may also be a crucial factor underlying variability between stores as some store may be freezing the chickens before their display for purchase by consumers. Pre-packaged chickens prepared for sale in stores contain moisture trapped in the packaging, which creates an environment that permits the survival of *Campylobacter* for several weeks at low temperatures [40]. Variation between stores at these stages of the route from the primary producers to consumers could therefore play a key role in the risk of infection for the public.

We also investigated the prevalence of *Campylobacter* virulence factor genes in the DNA of the *C*. *jejuni* isolates from fresh poultry in relation to their growth capacity. Multiple virulence factors linked to adherence, invasion capabilities and toxin production have been implicated in the ability of *Campylobacter* to colonize and cause disease, and the virulence genes selected for investigation in this study are all significantly linked to disease pathogenesis [41] As expected, *ciaB* was universally detected in all PCR-positive samples and all 5 virulence factors were present in 88.9% of the colonies on Karmali plates that proved to be *C*. *jejuni*. *C*. *jejuni* is known to utilize heat-shock proteins, including the ATP-dependent protease *clpP*, which repairs and prevents the damage caused by an accumulation of unfolded proteins [42, 43]. Its high prevalence among our isolates may be accounted for by the utility of this gene in helping *C*. *jejuni* survive through the high temperatures encountered during the summer time in the Arabian Gulf countries.

We detected high prevalence of *cadF* and *cdtB* in agreement with several studies that found it in nearly all of the *Campylobacter* isolates [44, 45, 46, 47]. All five virulence factors were expressed in a higher percentage when the sample was both Karmali culture-positive and PCR-positive for *C*. *jejuni*.

Another interesting finding was that virulence factors were expressed much more rarely in the *C*. *jejuni* DNA isolated from BPW-enriched media from chickens that did not produce colonies capable of growing on Karmali plates. This contrasts with the almost universal prevalence of some of the virulence factors in isolates that did grow successfully, highlighting the role of virulence factors in the fitness of *Campylobacter* isolates, and may well be linked to their ability to grow off the host. It is conceivable that the PCR-positive samples from BPW enriched media that failed to grow in Karmali plates were largely unable to do so because of the absence of the critical virulence factors that we screened for, and perhaps other as yet unknown genes that may also have been absent from these bacteria. The low prevalence of the virulence genes in the culture negative- PCR positives may be attributable also to these being other *Campylobacter* species or possibly other members of the *Campylobacterales* that lacked these genes but were nevertheless successfully detected by our PCR probes. The presence of multiple virulence genes is considered to enhance the invasiveness of *Campylobacter* but exactly how presence/absence of combinations of, or indeed any single, factor/s may be linked to *in vivo* virulence and pathogenicity in humans is still uncertain.

We found significant associations among the virulence factors identified on the Karmali-positive cultures. Positive cultures with *cadF* were almost always also positive for *clpP*, while *cdtB-*positive cultures had a high prevalence of *clpP* and *htrB*. Another study has shown increased invasiveness for a combination of *cdtB* and *ciaB* [48] but although we did not find a statistically significant interaction between these factors, all isolates were positive for *ciaB*, and since 94.9% also had the *cdtB* gene, there were very few isolates without this combination (only 5.1%). If the different combinations of virulence factors among *C*. *jejuni* isolates are linked to different potentials for causing disease, this may explain strain-specific difference in virulence [49] and some of the variation reported between studies. In future, it will be of interest to establish the *in vivo* pathogenicity of such strains circulating in Qatar, since this may be influenced by the host genetic composition and local environmental factors [5].

Finally, the detection of *Campylobacter* from skin samples of chickens has shown that the products being sold in Qatar have a high degree of contamination. It is pertinent also that these bacteria can control transcription of virulence genes that are necessary for survival under stressful conditions [50]. Thus, the detection of *Campylobacter* by culture may give false negative results if there are viable but non-culturable bacteria within the sample being investigated, and so the prevalence of *C*. *jejuni* can be easily underestimated. The failure of diagnostic procedures relying solely on culture methods to detect all *Campylobacter* means there is an increased chance of consumer exposure even if there is a screening program based on culture in place. To the best of our knowledge, there is no current screening for *Campylobacter* in Qatar and because *C*. *jejuni* is the most common cause of bacterial gastroenteritis, it is crucial to instigate a thorough and reliable monitoring program to reduce the availability of contaminated products sold in the supermarkets in Qatar. Our work has provided data for the first time on the prevalence of five virulence genes in the contaminated poultry sold in Qatar and has identified differences in the prevalence and in combinations of virulence genes in isolates that can and cannot grow in classic maintenance media. This may well provide a starting point for linking presence/absence of particular virulence genes with actual *in vivo* virulence and pathogenicity. Because of the relatively low infective doses of *Campylobacter* that are required to initiate infection in humans, it will be important to explore the relationships identified above between certain *Campylobacter* virulence genes and their ability to survive in poultry meat and hence contribute to the incidence of campylobacteriosis.

## Supporting Information

S1 File Summary of the data used during this analysis. (XLSB)
